# Interactive apps prevent gender discrepancies in early‐grade mathematics in a low‐income country in sub‐Sahara Africa

**DOI:** 10.1111/desc.12864

**Published:** 2019-06-23

**Authors:** Nicola J. Pitchford, Antonie Chigeda, Paula J. Hubber

**Affiliations:** ^1^ School of Psychology University of Nottingham Nottingham UK; ^2^ Chancellor College University of Malawi Zomba Malawi

**Keywords:** education, gender inequity, mathematics, reading, tablet technology

## Abstract

Globally, gender differences are reported in the early acquisition of reading and mathematics as girls tend to outperform boys in reading, whereas boys tend to outperform girls in mathematics. This can have long‐term impact resulting in an under‐representation of girls in Science, Technology, Engineering and Mathematics subjects. Recent research suggests that sociocultural factors account for differences across genders in the acquisition of these foundational skills. In this study, we investigated whether a new technology‐based intervention, that included activities accessible to both boys and girls, can reduce gender differences from emerging during the early primary school years. The novel instructional method used in this study employed apps developed by onebillion© delivered individually through touch‐screen tablets. Over a series of experiments conducted in Malawi, a low‐income country in sub‐Sahara Africa, we found that when children were exposed to standard pedagogical practice typical gender differences emerged over the first grade (Experiment 1). In contrast, boys and girls learnt equally well with the new interactive apps designed to support the learning of mathematics (Experiment 2) and reading (Experiment 3). When implemented at the start of primary education, before significant gender discrepancies become established, this novel technology‐based intervention can prevent significant gender effects for mathematics. These results demonstrate that different instructional practices influence the emergence of gender disparities in early mathematics. Digital interventions can mitigate gender differences in countries where standard pedagogical instruction typically hinders girls from acquiring early mathematical skills at the same rate as boys.

A video abstract of this article can be viewed at https://www.youtube.com/watch?v=55x-6hhAY9M&feature=youtu.be

**Research Highlights**

- Significant gender disparities emerge in mathematics and reading over the first grade of primary school in Malawi.
- Interactive apps that teach basic mathematics and reading are significantly more effective at raising early learning outcomes than regular, class‐based, teacher‐led, instruction.
- Boys and girls learn equally well with interactive apps designed to support the acquisition of early‐grade mathematics and early‐grade reading.
- When implemented at the start of primary education, interactive apps prevent gender discrepancies in mathematics.

## 1 INTRODUCTION

The United Nations 2030 Sustainable Development Goal 4 aims to “Ensure inclusive and equitable quality education and promote lifelong learning for all” (United Nations, 2016). Yet, gender differences in the acquisition of reading and mathematics are commonly reported in countries around the world. Globally, girls tend to underperform in mathematics compared to boys, but reading levels for girls are generally higher than those of boys (OECD, 2016; Saito, 2011; UNESCO, 2015). These gender disparities are often entrenched at a country level and appear resistant to intervention. Analysis of ten years of the Programme for International Student Assessment (PISA) data revealed that no country had successfully eliminated gender differences in both domains (Stoet & Geary, 2013). Whilst some biological accounts have been put forward (e.g., Burman, Bitan, & Booth, 2008; Halpern et al., 2007), most recent research implies sociocultural factors account for gender differences in reading and mathematics. For example, it has been suggested that during the preschool years, girls are likely to have more reading experience than boys because they are more motivated to read and mothers talk more to daughters than sons (Sigmundsson, Eriksen, Ofteland, & Haga, 2017), which supports the acquisition of oral language processing skills that are critical for learning to read (Nation & Snowling, 2004). Furthermore, Spelke (2005) argued that the composition of formal mathematics tests may favour boys over girls and sociocultural factors in the preschool environment in Kenya have been suggested to differentially influence the early acquisition of numerical concepts in boys and girls (Ngware, Ciera, Abuya, Oketch, & Mutisya, 2012). However, few gender differences in visuospatial processing abilities of very young children are reported in the literature (Spelke, 2005), which suggests that girls should have a similar propensity as boys to learn mathematics from an early age.

A recent study from the Netherlands supports this supposition. Hutchinson, Lyons, and Ansari (2018) applied Bayesian and frequentist analyses to data from large sample of 1,391 children aged 6–13 years and reported that an advantage for boys over girls in foundational numerical skills was the exception rather than the norm. Similarly, another recent study by Bakker, Torbeyns, Wijns, Verschaffel, and Smedt (2018) used a Bayesian approach to quantify the evidence in favour of gender differences compared to gender equality in preschool children's early mathematical skills. In this study, a group of 402 Belgian children aged 4–5 years were given eight numerical tasks (verbal counting, object counting, numeral recognition, symbolic comparison, nonsymbolic comparison, nonverbal calculation, number order, dot enumeration). Results showed that preschoolers' early numerical competencies were characterized by gender equality rather than gender discrepancies.

The latest PISA data (OECD, 2016) revealed that whilst girls consistently outperformed boys in reading at age 15 in all 72 participating countries, there was more variability in performance for mathematics. Overall, boys outperformed girls in mathematics, and a significant advantage for boys was found in 28 of the 72 countries (39%) that participated, whereas the reverse was found in only 9 countries (13%) where girls significantly outperformed boys. Similarly, the Southern & Eastern Africa Consortium for Monitoring Educational Quality (SACMEQ) III data for Grade 6 children (Saito, 2011) showed that boys outperformed girls in mathematics in 8 of the 15 countries (53%), whereas girls significantly outperformed boys in 1 (7%) country—Seychelles, which has comparatively low levels of societal gender inequity, as measured by the Gender‐related Developmental Index (Ngware et al., 2012).

Further evidence links sociocultural factors to gender disparities in mathematics. Dickerson, McIntosh, and Valente (2015) combined data of 50,000 African pupils from SACMEQ II and the Program for the Analysis of Education Systems databases. They reported that gender differences in mathematics were predicted by a country's fertility rate and the proportion of uneducated adult women. Similarly, an analysis of PISA results for 276,000 15‐year‐olds across 40 countries collected in 2003 showed gender differences in mathematics decreased as country‐level gender equality increased (Guiso, Monte, Sapienza, & Zingales, 2008), where gender equality was measured by the World Economic Forum's Gender Gap Index (Greig, Hausmann, Tyson, & Zahidi, 2006) which considers female education, well‐being, and economic and political opportunities. This was only apparent, however, for certain aspects of mathematics. For example, gender differences in geometry, believed to be largely spatial in nature, were not affected by sociocultural factors.

Levels of reading attainment are linked to a country's economic growth and to an individual's health, nutrition, rate of fertility and mortality, and income potential (Verner, 2005), but mathematics attainment has been shown to have a greater impact than reading on an individual's income potential (Crawford & Cribb, 2013; Dickerson et al., 2015; Geary, 2004). Findings from several African countries indicate an increase in mathematics test scores of only 0.1 standard deviations produces an increase in income of between 2% and 6.5% (Dickerson et al., 2015, p. 19). The gender disparity in mathematics observed in some countries globally puts girls at a disadvantage compared to boys in terms of future earnings potential and well‐being. Early interventions that support the acquisition of mathematical skills in girls are necessary to prevent a long‐term gender disparity from emerging at the disadvantage of girls. A change in instructional practice, from class‐based teacher‐led instruction, which might reinforce sociocultural biases, to individualized self‐paced instruction using app‐based technology, that include activities accessible to both boys and girls, may reduce the gender gap in mathematics that typically emerges over the primary school years.

### 1.1 Current study

This investigation focuses on the emergence of gender discrepancies in reading and mathematics over the early years of primary school in Malawi, a low‐income country in sub‐Sahara Africa, with a history of underachievement and gender inequalities throughout its education system (Kadzamira & Rose, 2003). By the end of primary school, less than 50% of Malawi children have achieved basic competency in reading and mathematics (Milner, Mulera, Banda, Matale, & Chimbo, 2011). Gender inequalities were first targeted through access to learning rather than attainment levels (e.g., Chimombo, 2009; Lewin & Sabates, 2012). However, with the adoption of the Sustainable Development Goals (United Nations, 2016), emphasis has shifted from the availability of education to the quality and equity of learning. Like other countries in the region, data from SACMEC 11 (2000) and SACMEC III (2007) show Malawian boys consistently outperformed girls at Grade 6 in mathematics (Saito, 2011). However, Malawian boys also outperformed girls in reading at Grade 6, a long‐term difference that has been reported since SACMEC I commenced in 1995 (Saito, 2011). A recent study by Mulera, Ndala, and Nyirongo (2017) showed that whilst the gender difference for reading reduced over age, such that by 22 years there was no observable difference in the mean reading level of males and females, the gender difference in mathematics widened with age. This suggests particular attention should be paid to educational interventions that prevent early gender differences in mathematics from emerging, as early learning experiences are a significant predictor of attainment at the end of primary school (Sylva, Melhuish, Sammons, Siraj‐Blatchford, & Taggart, 2010).

Over three experiments, we investigated differences in the performance of boys and girls on standardized measures of reading and mathematics in samples of children in the first three grades of the Malawi state‐funded primary school system. We first assessed gender inequalities in reading and mathematics attainment in 14 primary schools located across Malawi. We then investigated the learning of mathematics and reading through an innovative intervention that uses digital technology to see whether this new instructional method, that includes activities accessible to both boys and girls, can offer a potential solution for equating early learning opportunities of these core foundational skills and preventing gender differences from emerging.

### 1.2 Instructional design

The technology intervention employed in this study uses a set of interactive apps developed by the British not‐for‐profit, onebillion©, joint winners of the Global Learning XPRIZE*.* The apps are delivered to individual children through touch‐screen tablets connected to headphones. Designed to support mastery of basic mathematical and reading skills through a series of game‐like activities, the apps include a sequence of topics that are mapped to a well‐structured and staged early years curriculum (see Table 1). They capitalize on multisensory, child‐centred, playful learning and include a set of features that engage children in the learning process, for example interactive virtual manipulatives (e.g., moving objects around the screen), immediate task feedback (positive and negative) on every interaction with the technology and motivating rewards (e.g., big yellow tick and high‐pitched “ping” sound when successfully completing a task). A female in‐app teacher gives clear instructions and demonstrations in Chichewa, the language of instruction in Malawi, which can be repeated upon demand by the child. Children complete the activities independently, at their own pace. Learning is assessed through an in‐app quiz at the end of each topic, and a certificate is awarded for each topic successfully completed.

**Table 1:** Topics/units covered in the interactive apps developed by onebillion© designed to support the development of early‐grade mathematics and reading

Mathematics: 28 topics	Reading: 4 core units
Sorting and Matching Counting to 3 Lines and patterns Counting 4 to 6 Where is it? Counting 7 to 10 Patterns and shapes Counting 1 to 10 Comparing Adding and taking away Shape and position Counting to 20 Sharing More counting Telling the time Add and subtract Count in tens and fives How tall, how long? Count to 100 2D shapes Number lines Fractions Weigh it! More number work 3D shapes Measure time More + and ‐ How much can it hold?	*Phonemic awareness* Identification of “same” and “different” sounds (child picks out objects whose names start with, end with, or contain, specific phonemes), start with words, move on to syllables, and finish with individual phonemes. *From letters to words* Includes: meet the letters, make syllables, make words, and the alphabet. From introduction of single letters, moves on to graphemes with two or more letters, common nonphoneme consonant pairs and clusters, vowels, monosyllabic words, compound words, blending of phonemes and vowels to make syllables, blending of syllables to make word, letter names, letter–sound correspondences *Phrases, sentences, and paragraphs* Learn about reading from left to right, and from the first line down; the function of capital letters, and punctuation; assemble phrases and sentences from words, to match audio; practise high‐frequency words in context; develop morphological awareness, for example about how tenses and plurals are formed. *Stories* 80 + stories, graded into reading bands, each story can be presented in up to seven different story modes; some stories are specially written, to bring in familiar situations from everyday life; most are heavily illustrated.

Proof of concept for the onebillion maths app intervention used was first established by Pitchford (2015) through a pupil‐level Randomized Controlled Trial (RCT) involving 283 children from the first three grades of schooling of one urban primary school in Malawi. After 8 weeks of using the interactive maths apps, intervention children had significantly higher scores on experimental measures of conceptual mathematics knowledge (4% higher attainment, Cohen's *d* = 0.23) and mathematics curriculum knowledge (18% higher attainment, Cohen's *d* = 0.75) than control children who received regular classroom practice only. Differences in learning were attributed to the apps, not the tablet technology, as a placebo group that received interaction with the hand‐held tablets and design apps that required similar drag and drop movements of objects on the screen, did not improve learning outcomes. As this was the first time these children had used tablet technology to support their learning, the placebo group demonstrates that novelty and motivational factors alone were not responsible for increased learning gains. Importantly, girls learnt just as well as the boys when using the interactive maths apps suggesting that, if implemented at the start of primary school, this new educational technology could provide a solution for closing the gender gap in mathematics that typically emerges over the early primary school years.

**Figure 1:**
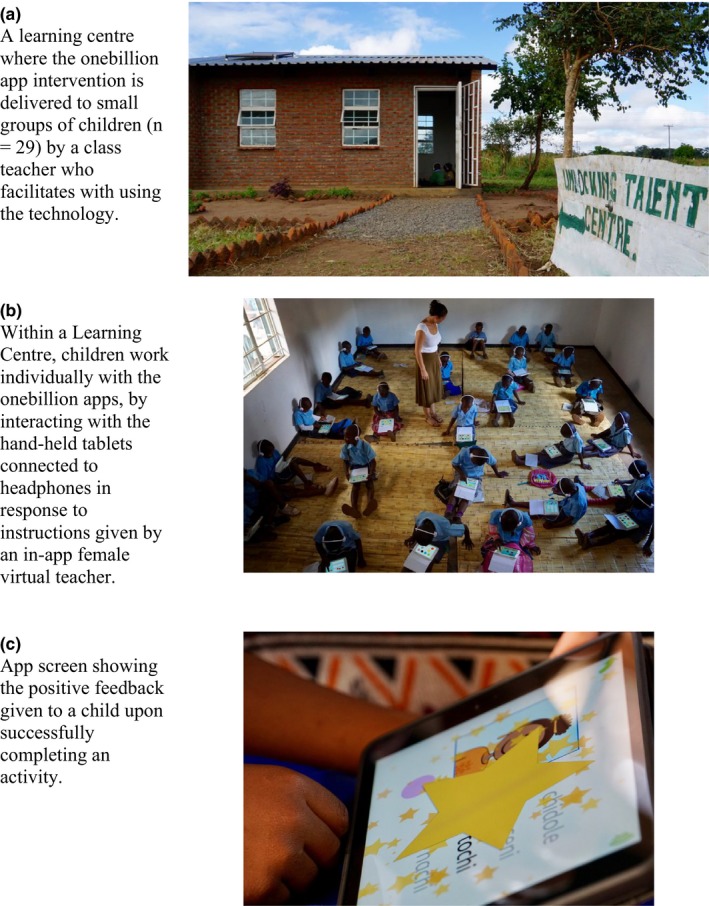
Illustration of how the onebillion app intervention is implemented in Malawian primary schools. Photos courtesy of www.onebillion.org

In an attempt to raise early learning outcomes nationally, the Malawi government is now rolling out this novel digital technology intervention to over 100 primary schools countrywide, in conjunction with the Voluntary Services Overseas (VSO). Each participating school is equipped with a set of 30 Apple iPad minis (29 for pupils and one for the teacher) loaded with the onebillion apps. The intervention is administered in a purpose‐built room, called a “learning centre”, by a classroom teacher who acts as a technology facilitator (see Figure 1). They register children on the iPads, solve technical issues, and monitor children's progress. The apps are very easy for children to use and require little support from teachers. Pedagogical input from the teacher is minimal.

We first report baseline data collected in 2015 with a large group of children attending one of 14 participating primary schools across Malawi. Thus, Experiment 1 established the impact of gender on the development of early‐grade reading and mathematics in Malawi through standard pedagogical instruction, prior to the introduction of the digital technology intervention. We then examined the effect of the digital technology intervention on gender differences in the learning of early‐grade mathematics (Experiment 2) and early‐grade reading (Experiment 3) by comparing performance gains over time for boys and girls who received the digital technology intervention to those who received standard classroom instruction only. As the software used to support the learning of early mathematics and reading was developed by onebillion, critical features of the app design and interface that might impact on attainment (Sung, Chang, & Liu, 2016) were the same across domains, hence controlling for any effect of these components from influencing results.

## 2 METHODS

### 2.1 Ethical approval

The National Commission for Science and Technology in Malawi granted ethical approval for this study. This was required by the School of Psychology, University of Nottingham, that abides by ethical guidelines specified by the British Psychological Society. Opt‐out parental consent was used to accommodate for the high rate of illiteracy in Malawi among the adult population. Opt‐in parental consent is not suitable in Malawi and would lead to a highly biased sample. According to standard ethical guidelines, meetings were held with the parent association at each school who were informed of the study and acted on behalf of the parents of participating children. Parents were informed of the study through the parent association and given the option not to include their child in the study. No parent chose to withdraw their child from the study. All data were password‐protected and accessible only to the research team.

### 2.2 Experiment 1

SACMEQ data show a consistent advantage for boys outperforming girls in both reading and mathematics (Saito, 2011) in Grade 6 Malawi pupils. This study was conducted to understand when these discrepancies become established by examining baseline data collected in November 2015.

#### 2.2.1 Participants

A sample of 1,217 children attending 1st or 2nd grade of 14 schools located across seven education districts in Malawi was recruited. In each district, one school had been assigned by the Malawi Ministry of Education, Science and Technology to receive the onebillion app intervention and one school was selected by the research team to act as a control school that delivered standard educational practice as specified by the national curriculum. Control schools were matched, as far as possible, to intervention schools on key demographics, including number of 1st‐ and 2nd‐grade pupils, number of 1st‐ and 2nd‐grade teachers, overall school size and geographical location, in an attempt to control for these variables influencing results. A prospective power analysis indicated that a sample of 1,200 pupils was required to detect an effect size of 0.5 for differences in scores between pupils receiving the intervention and pupils receiving standard classroom practice only, with 80% power and an intra‐cluster correlation of 0.25.

Descriptive statistics of the sample composition are given in Table 2. Children progress to the next school year based on their level of attainment rather than their age. Repetition rates are prevalent in the first four years of primary school (Maluwa‐Banda, 2004). It is common for a class to include children with a wide range of ages. Data were collected by an in‐country team of assessors from Invest in Knowledge, who were independent from the researchers analysing the data (first and third author). Data collection was managed in‐country by the second author. Random selection of children per gender occurred within each class at each school, as children lined up according to gender then the evaluators chose the first child in each line followed by every third child until the required sample size was reached. The numbers of girls and boys selected at each school were determined based on the gender ratio in each school grade. Table 1 reports the number and age of children included in the sample by grade and gender.

**Table 2:** Descriptive statistics for the sample of children participating in Experiment 1

Measure	Sample (*n* = 2017)			
	Grade 1 (*n* = 608)		Grade 2 (*n* = 609)	
	Boys	Girls	Boys	Girls
*n*	294	314	306	303
Age (years)				
Mean (*SD*)	6.66 (1.17)	6.42 (1.07)	8.43 (1.57)	7.93 (1.33)
Median	6	6	8	8
Min – Max	5–12	4–11	6–15	6–12
Reading				
EGRA % Mean (*SD*)	0.94 (1.44)	1.10 (1.78)	3.48 (6.30)	5.03 (8.74)
Mathematics				
EGMA % Mean (*SD*)	9.22 (6.78)	8.96 (6.58)	27.72 (16.35)	24.70 (16.20)

Note

Early‐grade reading and mathematics scores (per cent correct) at the start of the 2015–2016 school year.

#### 2.2.2 Assessments

Chichewa versions of the Early Grade Reading Assessment (EGRA, USAID, 2010) and Early Grade Mathematics Assessment (EGMA, USAID, 2011) were administered. These standardized assessments are commonly used in international comparison studies and are suitable for children aged 5–15 years. Standardized procedures were followed for administering and scoring the subtests of EGRA and EGMA. EGRA included subtests assessing letter naming (out of 100), syllable segmentation (out of 10), knowledge of initial sounds (out of 10), syllable reading (out of 100), familiar word reading (out of 50), nonword reading (out of 50), fluency in reading a written passage (out of 61), reading comprehension (out of 5), and listening comprehension (out of 5); total possible raw score = 391. EGMA included subtests assessing number identification (out of 10), discrimination between two quantities (out of 10), completing a pattern of numbers (out of 10), solving word problems (out of 4), and solving additions (out of 10) and subtractions (out of 10); total possible raw score = 54.

#### 2.2.3 Procedure

Children were assessed on an individual basis in a quiet area away from the rest of the class by the trained assessors. Order of presentation was counterbalanced across children to control for order effects in test administration. It took a maximum of 40 min per child to administer both assessments, which was usually completed in one session. When this was not possible, assessments were completed the following day. For each assessment, stimuli were presented to individual children on sheets of paper and the child's response was recorded by the evaluators using Samsung Galaxy tablets and Tangerine^TM^ software. The Data Manager at Invest in Knowledge downloaded and checked the data collected from the tablets and then sent the data to the researchers in password‐protected Excel and SPSS formats.

### 2.3 Experiment 2

To examine the impact of the onebillion maths app intervention on gender differences in early‐grade mathematics, we compared gains in mathematics scores at baseline in November 2015 to those obtained in January 2017 (when the sample was in grade 2), after the seven intervention schools had been implementing the onebillion maths app intervention for around three to six months. For the control group, we predicted an advantage in early‐grade mathematics would emerge for boys compared to girls over the first grade. In contrast, for the intervention group, if girls learn just as well as boys with the onebillion apps (Pitchford, 2015), we predicted that boys and girls would show similar improvement in mathematics attainment over the first grade after using the interactive apps, thus preventing a gender discrepancy from emerging.

#### 2.3.1 Participants

The original sample of 1st‐grade children reported in Experiment 1 took part in Experiment 2. This consisted of 608 pupils at baseline (see Table 2). However, the Malawi education system suffers from high rates of drop out and absenteeism (Lewin & Sabates, 2012) and by January 2017, when Experiment 2 was conducted, 58% of the original sample were not present in school. Thus, the final sample consisted of 256 children who were available for assessment. Descriptive statistics for this sample are reported in Table 3. Within this sample, 149 children had received the onebillion maths app intervention and 107 children were in a control group who had received regular, class‐based, teacher‐led instruction. At baseline, the final sample did not differ significantly across the control and intervention groups in age, *t*(254) = 0.61, *p* = 0.543, mathematics attainment (EGMA % correct), *t*(254) = 1.09, *p* = 0.276 or gender distribution, *x* ^2^(1, *n* = 256) = 0.03, *p* = 0.859, demonstrating the two instruction groups were well matched before the new maths app intervention was introduced.

**Table 3:** Descriptive statistics for the final sample of children participating in Experiment 2

Measure	Group Sample (*n* = 256)			
	Intervention (*n* = 149)		Control (*n* = 107)	
	Boys	Girls	Boys	Girls
*n*	61	88	45	62
Age (years)				
Mean (*SD*)	6.82 (1.13)	6.58 (1.15)	6.98 (1.16)	6.65 (1.18)
Median	7	6	7	6
Min–Max	5–11	5–11	6–11	5–11
Mathematics				
EGMA % Gain Mean (*SD*)	19.95 (15.11)	20.86 (16.14)	17.05 (16.12)	11.90 (12.61)
Between‐group effect size				
(intervention vs. control) Cohen's *d*	Boys = 0.186 Girls = 0.619			

Note

Mathematics gains (per cent) and between‐group effect sizes by gender across the 14‐month intervention period reported.

#### 2.3.2 Assessment

EGMA was administered at both assessment points according to standardized instructions as described in Experiment 1.

#### 2.3.3 Procedure

During the period between the two assessments of mathematical attainment, children in the control group received their usual mathematics instruction only, delivered by class teachers, in accordance with the National Primary Curriculum of Malawi. In contrast, children in the intervention group received mathematics instruction through the new digital technology intervention, that uses interactive maths apps delivered to individual children on an iPad mini connected to headphones, in the learning centre of their school. Each child received several 30‐min sessions with the interactive apps. The total mean time of interaction with the apps across the sample of intervention children was 8.9 hr (*SD* = 6.4), which is approximately eighteen 30‐min sessions per child across the 14‐month assessment period. Time with the app intervention was restricted by delays in building learning centres, hardware shortages, and timetabling constraints. Accordingly, on days when children in the intervention group could not access the learning centre, they received class‐based, teacher‐led, mathematics instruction, as per the control group.

### 2.4 Experiment 3

For the first time, we examined the effectiveness of a new interactive, child‐centred app, developed by onebillion^©^, to support the acquisition of early reading skills in Chichewa, the language of instruction in Malawi primary schools. As this newly developed app had not been trialled previously, we conducted a pupil‐level RCT in two primary schools to establish proof of concept for its effectiveness at supporting reading acquisition for boys and girls drawn from the first three grades, when reading instruction is given as part of the national curriculum.

We first examined gender differences in our sample at baseline (pre‐test) to determine if the global pattern of girls attaining higher reading scores than boys existed in our data. The reading app intervention was then implemented for 14 weeks to a group of children randomly selected from two primary schools that did not take part in Experiments 1 and 2. At the end of the intervention period, we compared gains in reading scores between pre‐test and post‐test for children using the new reading app to those achieved by control children who received standard classroom instruction only. We predicted gender differences would be found to advantage girls prior to the introduction of the new reading app. If gender differences in reading are influenced by sociocultural factors, then boys might be expected to learn just as well as girls with the new reading app.

#### 2.4.1 Participants

360 children from two Malawi primary schools took part in this pupil‐level RCT. Neither of the schools were part of Experiments 1 and 2 and were not using the maths apps when Experiment 3 took place. 180 children were randomly selected from each school to take part in the study: 60 children from each of grades 1, 2 and 3. In each grade, 30 children were randomly allocated to the control group and 30 were randomly assigned to the intervention group. The gender distribution was equal across grade and instructional group. Children were randomly selected and allocated to group by the research team, using random number generation, from class registers provided by VSO. All children were assessed at baseline (pre‐test). However, at post‐test, 40 children were not present in school on the day the assessments took place: 16 children from grade 1, 14 from grade 2 and 10 from grade 3. Composition of the study sample at each stage of the RCT is reported in Table 4. Descriptive statistics for the 320 children assessed at post‐test are reported in Table 5.

**Table 4:** CONSORT table describing composition of the sample at each stage of the pupil‐level randomized control trial for each of the three grades (G) that took part in Experiment 3

Study phase	Number of children	
Enrolment		
Eligible (all children in S1–S3)	850 (G1 = 347; G2 = 312; G3 = 191)	
Randomized (to one of two groups)	360 (G1 = 120; G2 = 120; G3 = 120)	
Excluded (due to group size study design constraints)	490 (G1 = 227; G2 = 192, G3 = 71)	
Allocation		
Group	Instruction with reading app	Instruction as usual
Randomized to group, pre‐tested and received intervention	180 (G1 = 60; G2 = 60; G3 = 60)	180 (G1 = 60; G2 = 60; G3 = 60)
Follow‐up		
Post‐tested	162 (G1 = 51; G2 = 53; G3 = 58)	158 (G1 = 53; G2 = 53; G3 = 52)
Lost to follow‐up (i.e., absent or transferred school by post‐test)	18 (10%) (G1 = 9; G2 = 7; G3 = 2)	22 (12.2%) (G1 = 7; G2 = 7; G3 = 8)
Analysed		
Final sample (present at pre‐test and post‐test)	162 (G1 = 51; G2 = 53; G3 = 58)	158 (G1 = 53; G2 = 53; G3 = 52)

**Table 5:** Descriptive statistics for the final sample of children participating in Experiment 3. Reading gains (per cent) and between‐group effect sizes by gender across the 14‐week intervention period reported

Measure	Group Sample (*n* = 320)			
	Intervention (*n* = 162)		Control (*n* = 158)	
	Boys	Girls	Boys	Girls
*n*	75	87	84	74
Age (years)				
Mean (*SD*)	7.87 (1.55)	7.43 (1.27)	7.95 (1.49)	7.57 (1.30)
Median	8	7	8	7
Min–Max	6–12	5–11	6–11	6–11
Reading				
EGRA % Gain Mean (*SD*)	8.32 (15.74)	10.65 (15.11)	4.29 (7.73)	5.09 (7.33)
Between‐group effect size				
(intervention vs. control) Cohen's *d*	Boys = 0.325 Girls = 0.460			

#### 2.4.2 Assessments

Reading attainment was assessed individually with children at pre‐test and at post‐test using EGRA, as described in Experiment 1.

#### 2.4.3 Procedure

EGRA was administered to individual children at pre‐test in January 2017, immediately before the reading app intervention was implemented. Assessments were carried out by a team of 21 trained evaluators from an independent company in Malawi, Research and Business Consult Ltd. Evaluators were blind to group allocation of children taking part in the trial.

The digital technology intervention was implemented by class teachers serving as technology facilitators in the learning centre of their school (see Figure 1) for 14 consecutive school weeks, excluding holidays. During the intervention period, children assigned to the intervention group received instruction with the new reading app for 60 min a day (maximum possible time with app = 70 hr). For all 180 intervention children in each school to attend daily sessions in the learning centre, intervention children missed some standard class‐based lessons, including teacher‐led reading instruction, as well as other subjects, because of timetabling and hardware constraints. However, if a regular reading lesson was being delivered in class when intervention children were present, they participated in that lesson, as per the rest of the class. Accordingly, the intervention children received some standard teacher‐led reading instruction, as well as individualized reading instruction with the reading app. Control children only received class‐based, teacher‐led, reading instruction, as is standard practice in Malawi primary schools.

## 3 RESULTS

### 3.1 Experiment 1

**Figure 2:**
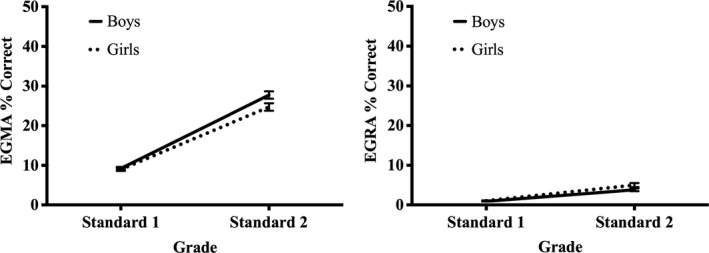
Attainment in mathematics (EGMA % correct, left panel) and reading (EGRA % correct, right panel) for boys and girls in grades 1 and 2 at baseline (November 2015) in Experiment 1. Error bars represent standard error of the mean

To enable direct comparison of gender differences in early‐grade reading and early‐grade mathematics, an overall score for percentage correct (i.e., the sum of the number of correct answers achieved on each subtest divided by the total possible score for all subtests) was calculated for each child for EGRA and EGMA. Descriptive statistics for reading (EGRA) and mathematics (EGMA) by grade and gender are reported in Table 2 and shown in Figure 2.

To investigate gender differences in reading and mathematics for children in the first two grades of primary school in Malawi, a 2 (Grade: 1, 2) × 2 (Gender: boy, girl) × 2 (Domain: Reading, Mathematics) mixed analysis of variance (ANOVA) was conducted, where Grade and Gender were between‐subject variables and Domain was a within‐subject variable. Results are reported using Bonferroni corrections for multiple comparisons.

Significant main effects were found for Grade, *F*(1, 1,213) = 472.89, *p* < 0.001, *η_p_* ^2^ = 0.298, as children in grade 2 achieved higher attainment scores than children in grade 1, and Domain, *F*(1, 1,213) = 2,240.54, *p* < 0.001, *η_p_* ^2^ = 0.649, as scores for mathematics were significantly greater than those for reading, but not for Gender, *F*(1, 1,213) = 1.14, *p* = 0.286, *η_p_* ^2^ = 0.001. However, there was a significant interaction between Gender and Domain, *F*(1, 1,213) = 13.50, *p* < 0.001, *η_p_* ^2^ = 0.011. Simple main effects showed girls scored significantly higher than boys overall for reading, *F*(1, 1,213) = 4.57, *p* = 0.033, *η_p_* ^2^ = 0.004, whereas boys scored significantly higher than girls overall for mathematics, *F*(1, 1,213) = 5.29, *p* = 0.022,, *η_p_* ^2^ = 0.004.

There was also a significant interaction between Domain and Grade, *F*(1, 1,213) = 2.86, *p* ≤ 0.001, *η_p_* ^2^ = 0.280, as the increase in scores between grade 1 and grade 2 was significantly greater for mathematics than reading. The interaction between Gender and Grade was not significant, *F*(1, 1,213) = 0.92, *p* = 0.337, *η_p_* ^2^ = 0.001, but the three‐way interaction between Gender, Domain and Grade was *F*(1, 1,213) = 9.08, *p* = 0.003, *η_p_* ^2^ = 0.007. Pairwise comparisons showed that for grade 1, there was no significant difference between boys and girls in reading, *F*(1, 1,213) = 0.13, *p* = 0.721, *η_p_* ^2^ = 0.000, or mathematics, *F*(1, 1,213) = 0.07, *p* = 0.799, *η_p_* ^2^ = 0.000. In grade 2, however, girls scored significantly higher than boys for reading, *F*(1, 1,213) = 7.12, *p* = 0.008, *η_p_* ^2^ = 0.006 and boys scored significantly higher than girls for mathematics, *F*(1, 1,213) = 9.00, *p* = 0.003, *η_p_* ^2^ = 0.007. For both reading and mathematics, a medium gender effect size was found in grade 2, although the direction of effect differed across domains. (Note: The standard deviations for EGRA and EGMA scores were not equal across Grade (see Table 1), so we conducted a separate 2 (Gender: boy, girl) × 2 (Domain: reading, mathematics) mixed ANOVA for each grade to discover whether the same pattern of results would be produced. Results from these two ANOVAs confirmed the findings reported above, demonstrating effects are robust to variation in standard deviations across Grade.).

### 3.2 Experiment 2

**Figure 3:**
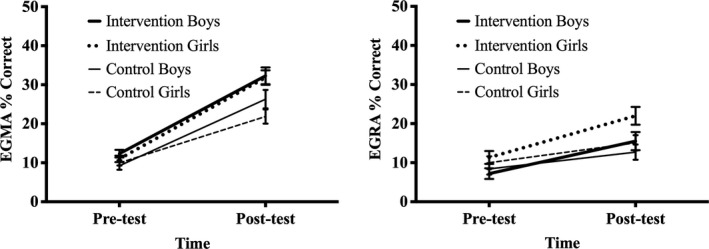
Pre‐test (baseline) and post‐test attainment in mathematics (EGMA % correct) and reading (EGRA % correct) for boys and girls receiving the new digital technology intervention for mathematics (Experiment 2, left panel) and reading (Experiment 3, right panel) compared to control children receiving standard class‐based, teacher‐led, instruction only. Error bars represent standard error of the mean

To assess the impact of the onebillion maths app intervention on attainment in early‐grade mathematics, an overall percentage correct score for EGMA was calculated for each child, for each assessment point, and then, a difference score for attainment across January 2017 and November 2015 was determined. Descriptive statistics for gains in mathematics by gender and group are given in Table 3. Figure 3 shows attainment levels in mathematics (EGMA percentage correct) across the 14‐month study period for boys and girls in the intervention and control groups.

To investigate differences in gains in mathematics between boys and girls receiving the maths apps and control children receiving normal classroom practice only, a 2 (Group: control, intervention) by 2 (Gender: boy, girl) between‐group ANOVA was conducted with difference scores (gains in mathematics across time) as the dependent variable. EGMA shows good reliability of 0.88 (USAID, 2011) so it is acceptable to use difference scores in this analysis (Trafimow, 2015). Results are reported using Bonferroni correction for multiple comparisons.

A significant main effect of Group was found, *F*(1, 252) = 9.34, *p* = 0.002, *η_p_* ^2^ = 0.036, as intervention children achieved greater gains in mathematics than control children across the 14‐month period. Neither the main effect of Gender, *F*(1, 252) = 1.20, *p* = 0.275, *η_p_* ^2^ = 0.005 or the interaction between Group x Gender, *F*(1, 252) = 2.44, *p* = 0.120, *η_p_* ^2^ = 0.010 was significant. Planned comparisons were conducted to investigate gender differences in response to type of instructional practice. As predicted, within the control group, boys achieved significantly greater gains in mathematics than girls, *F*(1, 252) = 3.04, *p* = 0.042, *η_p_* ^2^ = 0.012, one‐tailed (*p* = 0.083, two‐tailed). In contrast, there was no significant difference between gains in mathematics for boys and girls in the intervention group, *F*(1, 252) = 0.13, *p* = 0.719, *η_p_* ^2^ = 0.001.

### 3.3 Experiment 3

To explore how boys and girls learned with the new reading app, an overall percentage correct score for EGRA was calculated (i.e., the sum of the number of correct answers on each subtest divided by the total possible score for all subtests) for each child at pre‐test and post‐test; see Figure 3. To investigate the presence of gender differences in the sample of children at pre‐test, before the new reading app intervention was implemented, a 2 (Gender: boy, girl) x 3 (Grade: 1, 2, 3) mixed ANOVA was conducted with pre‐test EGRA percentage correct score as the dependent variable. Results are reported using Bonferroni corrections for multiple comparisons.

A significant main effect of Gender, *F*(1, 314) = 5.25, *p* = 0.023, *η_p_* ^2^ = 0.627 and Grade, *F*(1, 314) = 62.22, *p* < 0.001, *η_p_* ^2^ = 1.000 was found. Overall, girls achieved significantly higher reading scores at pre‐test than boys. Pairwise comparisons showed children in grade 3 had significantly higher pre‐test reading scores than children in grades 1 and 2 (both *p* < 0.001). However, there was no significant difference between the pre‐test reading scores of children in grades 1 and 2 (*p* = 1.00), and the interaction between Gender and Grade was not significant, *F*(1, 314) = 2.61, *p* = 0.075, *η_p_* ^2^ = 517.

Next, a difference score for attainment across the 14‐week intervention period was determined for each child (i.e. EGRA percentage gain). Descriptive statistics are reported in Table 5. To investigate the effect of gender on gains in reading attainment between children receiving instruction with the new reading app compared to children receiving normal class‐based, teacher‐led, instruction only, a 2 (Group: control, intervention) × 2 (Gender: boy, girl) between‐group ANOVA was conducted with reading gain percentage as the dependent variable. Grade was not entered into this analysis to retain statistical power and because the interaction between Grade and Gender was not significant at pre‐test. EGRA shows good reliability of 0.90 (USAID, 2010), so it is acceptable to use difference scores in this analysis (Trafimow, 2015). Results are reported using Bonferroni corrections for multiple comparisons.

There was a significant main effect of Group, *F*(1, 316) = 12.36, *p* = 0.001, *η_p_* ^2^ = 0.038, as reading gains were greater for intervention than control children. Neither the main effect of Gender, *F*(1, 316) = 1.31, *p* = 0.253, *η_p_* ^2^ = 0.004, or the interaction between Group and Gender, *F*(1, 316) = 0.31, *p* = 0.576, *η_p_* ^2^ = 0.001, was significant. As illustrated in Figure 3, similar learning gains were observed for boys and girls following instruction with the new reading app (mean EGRA percentage gain: boys = 8.32%, *SD* = 1.82, girls = 10.65%, *SD* = 1.62) and with standard class‐based reading instruction (mean EGRA percentage gain: boys = 4.29%, *SD* = 0.90, girls = 5.09%, *SD*=0.80).

## 4 DISCUSSION

This study investigated the impact of individualized instruction with interactive apps on learning attainment and gender inequalities in mathematics and reading during the early grades of primary school in Malawi. Experiment 1 demonstrated that children in the 1st grade of primary school in Malawi have very low attainment in reading and mathematics and ability is similar for boys and girls. However, 2nd grade, as performance levels increase, significant gender differences emerge, that advantage girls in reading and boys in mathematics. This is consistent with the global pattern of gender differences for reading and mathematics (OECD, 2016; Saito, 2011; UNESCO, 2015). It reinforces SACMEQ data for Grade 6 pupils in Malawi for mathematics and reveals that the significant gender effect advantaging boys is established as early as 2nd grade. However, our finding that girls significantly outperformed boys in reading at grade 2 is inconsistent with SACMEC data for Grade 6 pupils in Malawi, which shows an advantage for boys over girls in reading. Our results indicate a shift in gender effects for reading since SACMEQ III was conducted in 2007, perhaps in response to government efforts to improve primary education for girls, or suggest an early advantage for girls in reading is eradicated by Grade 6. SACMEQ IV data, which were collected between 2012 and 2014, will shed light on this finding, but results are currently not available.

Results from Experiment 2 demonstrated that the use of interactive, child‐centred, maths apps across the 1st and 2nd grade of primary school in Malawi can protect girls from falling behind boys at learning early‐grade mathematics. Despite a brief time using the onebillion maths apps, of just eighteen 30‐min sessions on average across the 14‐month study period, girls' attainment in mathematics was similar to that of boys. In contrast, for usual, class‐based, teacher‐led instruction, girls started to lag behind boys in learning mathematical skills. The lack of a significant Group by Gender interaction was most likely due to low statistical power in the sample, due to the high attrition rate between baseline (Experiment 1) and end‐line (Experiment 2) assessments. However, planned comparisons confirmed our predictions that a gender difference would emerge within the control group (significant on a one‐tailed test in the predicted direction), whereas girls and boys would learn equally well in the intervention group receiving instruction through interactive apps (Pitchford, 2015). Accordingly, the between‐group effect size for gains in mathematics across the intervention and control groups was small for boys (Cohen's *d* = 0.186) but relatively large for girls (Cohen's *d* = 0.619). This implies that gender disparities typically observed in acquiring early mathematical skills arise, to some extent, from sociocultural factors within the usual school and/or home environment.

Results from the pupil‐level RCT conducted in Experiment 3 provide proof of concept for the effectiveness of a new interactive app designed to support the acquisition of early‐grade reading skills in Malawi. Children from across the first three grades of primary school achieved significantly higher gains in reading when given instruction with the interactive reading app compared to standard, class‐based, teacher‐led practice only. As with early‐grade mathematics (Pitchford, 2015; Experiment 2), this demonstrates that digital technology interventions that utilize high‐quality, curriculum‐based, interactive apps can effectively raise attainment in early‐grade reading significantly more than standard practice. In the current study, this might reflect the extra time children spent learning to read with the app compared to standard pedagogical practice. However, a recent study in the UK, with the same maths apps as used in Malawi only delivered in English, showed the apps to be significantly more effective at raising mathematical attainment in the first year of schooling than standard class‐based practice even when the time spent learning mathematics was equated across both instructional groups (Outhwaite, Faulder, Gulliford, & Pitchford, 2018).

The effect of gender when learning to read with the new interactive reading app was also explored. Prior to the introduction of the new reading app, girls achieved significantly higher reading scores than boys at pre‐test, thus replicating results of Experiment 1. This corroborates the global gender difference for reading but contradicts previous SACMAQ data for Malawi which shows a consistent advantage for boys over girls in reading attainment at Grade 6. However, in contrast to Experiment 1, the interaction between Gender and Grade in Experiment 3 was not significant. This could indicate that an advantage for girls in reading was becoming established over the first term of schooling for 1st‐grade children. When baseline data were collected in January (2017) for Experiment 3, the mean difference was 1.09% correct on EGRA in favour of girls, whereas when baseline data were collected in November (2015) for Experiment 1, the mean difference was just 0.16%. Furthermore, whilst grade 2 scores were significantly higher than grade 1 scores in Experiment 1, there was no significant difference between reading levels of 1st‐ and 2nd‐grade children in Experiment 3. Comparing across Experiments 1 and 3, it can be seen that performance levels at baseline were similar for 2nd‐grade children (boys: Experiment 1 Mean = 3.84 (*SD* = 6.30); Experiment 3 Mean = 4.32 (*SD* = 5.09), *t*(484) = 0.87, *p* = 0.385, girls: Experiment 1 Mean = 5.03 (*SD* = 8.74); Experiment 3 Mean = 5.04 (*SD* = 6.68), *t*(481) = 0.01, *p* = 0.990). In contrast, for 1st‐grade children, baseline performance levels were significantly higher in Experiment 3 than Experiment 1 (boys: Experiment 1 Mean = 0.94 (*SD* = 1.44); Experiment 3 Mean = 2.78 (*SD* = 7.49), *t*(484) = 4.17, *p* < 0.0001, girls: Experiment 1 Mean = 1.10 (*SD* = 1.78); Experiment 3 Mean = 3.87 (*SD* = 8.51), *t*(481) = 5.47, *p* < 0.0001). This is most likely to reflect the introduction of the National Reading Program in grade 1 across Malawi at the start of the 2016–2017 school year. The Malawi government adopted the National Reading Program in a strategic attempt to raise early reading attainment in 1st‐grade children across the country. It is administered at the class level by teachers who have been specially trained to deliver this prescriptive, instructional programme, that is based on evidence from the psychology of learning to read. These results are a preliminary indication that the Malawi National Reading Program is effective at raising reading levels in 1st‐grade children. Findings from a national trial are forthcoming.

When gains in reading scores across the intervention period were compared, neither the main effect of Gender nor the interaction between Gender and Group was significant. This implies that over the 14‐week study period, progress in reading was similar for boys and girls for both instructional practices, although attainment levels at post‐test were significantly higher for both genders when given instruction with the interactive reading app. Accordingly, between‐group effect sizes were similar in magnitude at post‐test for boys (Cohen's *d* = 0.325) and girls (Cohen's *d* = 0.460) and illustrated a medium effect of learning to read with the new interactive app compared to standard, class‐based, teacher‐led, instruction.

Thus, for both domains, instruction with interactive apps significantly raised early learning outcomes compared to standard pedagogical practice. Introducing digital technology interventions within primary education in Malawi could thus be an effective way to raise early educational attainment in core foundational skills. The use of interactive apps to support the acquisition of reading and mathematics could also equalize learning opportunities for boys and girls. With standard pedagogical instruction, significant gender differences emerged across 1st grade, that benefitted girls in reading and boys in mathematics, consistent with the global pattern frequently reported in the literature (Dickerson et al., 2015; OECD, 2016; Saito, 2011; UNESCO, 2015). In contrast, similar learning gains were found for boys and girls in both domains when given instruction with interactive apps. These findings corroborate recent research that suggests gender equality is the norm for early mathematics prior to school entry (Bakker et al., 2018; Hutchinson et al., 2018) and indicate that sociocultural factors are responsible for the emergence of gender differences in the early grades of primary schools in Malawi.

For mathematics, standard, class‐based, teacher‐led, instruction resulted in boys achieving significantly higher performance levels in early‐grade mathematics than girls across the 1st grade (Experiments 1 and 2), but introducing the interactive apps at the start of the first year of school prevented a gender discrepancy in mathematics from emerging. This suggests that sociocultural factors typically operate within the school and/or home environment to advantage boys in learning early mathematical skills. Our data show that introducing digital technology interventions that are known to be effective at supporting the acquisition of early mathematics, and include activities accessible to both boys and girls, into an education system with a long history of underachievement and gender disparity can raise learning outcomes and close the gender gap in the early primary school years. Although gender differences in mathematics are most prevalent in countries with large rates of fertility and uneducated women, such as Malawi (Dickerson et al., 2015), these results indicate that gender inequity linked to these sociocultural factors can be overcome by changes in instructional regime. Notably, these results were achieved from a reasonably small time with the intervention, as children used the interactive maths apps for a total of only nine hours on average across a 14‐month period. Increasing time of task resulted in greater learning gains when these maths apps were delivered in English to children in British primary schools (Outhwaite, Gulliford, & Pitchford, 2017). Prolonged usage of these apps in Malawian primary schools should therefore enhance learning outcomes over time for girls and boys.

Results for reading were more complex. A significant gender effect emerged across the first year of schooling to the advantage of girls with standard classroom instruction (Experiment 1), but a significant advantage in reading was found for girls in Experiment 3 at pre‐test, before the introduction of the reading app. As girls and boys learnt to read equally well with the interactive reading app and standard class‐based reading instruction across the 14‐week reading trial, the gender discrepancy persisted for reading for both intervention and control children. This supports Logan and Johnston (2010) who noted that girls consistently outperform boys at reading regardless of type of instruction and the structure of the language in which they are learning to read. Accordingly, this might indicate a strong biological basis for learning to read that advantages girls (e.g., Burman et al., 2008). However, Experiment 3 reported on children across the first three grades of primary school and baseline data were collected in January, four months after the start of the school year. To test whether interactive apps can prevent a gender difference from emerging for early‐grade reading, the reading app used in this study would need to be introduced at the start of primary education, in 1st grade, before a gender difference begins to emerge, to assess its impact on reading attainment for boys and girls at the start of 2nd grade.

Time on task and attendance data were not available for Experiments 2 and 3. Also, it was it possible to observe the extent to which teachers implemented the apps as intended, or how maths and reading was being taught via more conventional teacher‐led techniques in the control groups. These limitations need to be addressed in future work, to understand whether additional benefits of using these apps might arise that impact children's willingness to go to school, and inform best practice for teaching early‐grade mathematics and reading in Malawi, with or without digital technology.

The interactive apps used in this study were created by the same software publisher so the structure and interface of the apps were very similar across domains. Children received instructions from a virtual female teacher, could repeat the instructions as often as required, received feedback on every interaction with the apps, and were required to pass a quiz on completion of each learning unit in order to progress to the next unit. These apps integrate active, engaged, meaningful, and socially interactive learning with a specific learning goal (Hirsh‐Pasek et al., 2015) and combine the benefits of direct instructional approaches (Kirschner, Sweller, & Clarke, 2006), in particular, a stepped curriculum, rehearsal, contingent feedback, and rewards, with aspects of free play (Gray, 2015), for example, choice, self‐regulation, and control. The features embedded within these apps are likely to facilitate learning for both genders, resulting in similar learning gains for boys and girls (Outhwaite et al., 2018; Pitchford, 2015). Future research could explore if some of these app features are more important than others in promoting learning outcomes. This might help to differentiate mixed findings on the effectiveness of app‐based interventions in supporting early learning (Haβler, Major, & Hennessy, 2015). What is clear is that the combination of features embedded in the onebillion**©** apps is equally effective at raising early mathematics and reading skills for girls and boys. This suggests that other apps that embody the same range of features should also be effective at mitigating gender differences in early education.

In contrast, progress differed across genders with standard classroom practice, indicating that sociocultural factors impact on girls' and boys' early education differentially (Spelke, 2005). It has been argued that girls are more motivated to learn to read than boys and motivation predicts later reading success (Logan & Johnston, 2010), anxiety of female teachers when teaching mathematics adversely impacts girls but not boys (Plante, Protzko, & Aronson, 2010), and girls prefer supportive learning environments whilst boys prefer competitive ones (Ngware et al., 2012). The apps used in this study are highly motivating and supportive, due to the game‐based activities and continual feedback and certificates awarded to the child when they have successfully completed a learning topic. As children work independently, at their own pace, there is little scope for competition between children. This is in stark contrast to the usual primary school classroom in Malawi, where in excess of 80 pupils can by vying for the attention of one teacher (World Bank, 2010). Accordingly, it is unlikely that enhanced teacher training alone could alleviate the range of sociocultural factors that appear to contribute towards gender discrepancies emerging in early education that the onebillion© apps seemingly overcome. Enhanced teacher training could be effective at raising awareness of how gender differences emerge and the importance of overcoming explicit and implicit biases in early education. These biases might affect access to technology interventions, as teachers may hold stereotypes of which children will most benefit from or enjoy using technology, and allocate devices accordingly. Access to the app technology was controlled for in our experiments, but in everyday practice teacher stereotypes may influence access to the technology. Measures should be taken to ensure the intervention is delivered as intended, through rigorous and regular monitoring, to enable boys and girls equal access to the apps.

Our results are particularly promising for mathematics as they suggest that if embedded within a country at the start of primary education, interactive apps that teach mathematics in the early grades could lead to enhanced attainment in mathematics for girls in later education, which may impact long term on girls pursuing Science, Technology, Engineering and Mathematics (STEM) subjects (Cheryan, Ziegler, Montoya, & Jiang, 2017; Mpuchane, 2011). Kultuerl‐Konak, D'Allegro, and Dickinson (2011) noted that teaching style influenced subject choices made by girls and recommended teaching STEM subjects in a way that facilitates learning rather than simply instructing. Interactive apps allow children to learn by exploration, as they receive constant feedback on their actions, which they are able to execute at their own pace. This is an important development for mathematics education and potentially offers a global solution for equality of mathematical attainment in the early years. If interactive apps can prevent gender differences from materializing at the start of a child's education, they may help provide an equal foundation for learning mathematics in the future.

## CONFLICT OF INTEREST

The authors declare no conflict of interest with the work reported in this article. The work was carried out in the absence of any personal, professional or financial relationships that could potentially be construed as a conflict of interest.

## DATA AVAILABILITY STATEMENT

The data that support the findings of this study are available from Professor Nicola Pitchford upon reasonable request.
